# Adaptor protein-3: A key player in RBL-2H3 mast cell mediator release

**DOI:** 10.1371/journal.pone.0173462

**Published:** 2017-03-08

**Authors:** Elaine Zayas Marcelino da Silva, Edismauro Garcia Freitas-Filho, Devandir Antonio de Souza-Júnior, Luis Lamberti Pinto daSilva, Maria Celia Jamur, Constance Oliver

**Affiliations:** Department of Cell and Molecular Biology and Pathogenic Bioagents, Ribeirão Preto Medical School – University of São Paulo, Ribeirão Preto, São Paulo, Brazil; Medical College of Wisconsin, UNITED STATES

## Abstract

Mast cell (MC) secretory granules are Lysosome-Related Organelles (LROs) whose biogenesis is associated with the post-Golgi secretory and endocytic pathways in which the sorting of proteins destined for a specific organelle relies on the recognition of sorting signals by adaptor proteins that direct their incorporation into transport vesicles. The adaptor protein 3 (AP-3) complex mediates protein trafficking between the trans-Golgi network (TGN) and late endosomes, lysosomes, and LROs. AP-3 has a recognized role in LROs biogenesis and regulated secretion in several cell types, including many immune cells such as neutrophils, natural killer cells, and cytotoxic T lymphocytes. However, the relevance of AP-3 for these processes in MCs has not been previously investigated. AP-3 was found to be expressed and distributed in a punctate fashion in rat peritoneal mast cells *ex vivo*. The rat MC line RBL-2H3 was used as a model system to investigate the role of AP-3 in mast cell secretory granule biogenesis and mediator release. By immunofluorescence and immunoelectron microscopy, AP-3 was localized both to the TGN and early endosomes indicating that AP-3 dependent sorting of proteins to MC secretory granules originates in these organelles. ShRNA mediated depletion of the AP-3 δ subunit was shown to destabilize the AP-3 complex in RBL-2H3 MCs. AP-3 knockdown significantly affected MC regulated secretion of β-hexosaminidase without affecting total cellular enzyme levels. Morphometric evaluation of MC secretory granules by electron microscopy revealed that the area of MC secretory granules in AP-3 knockdown MCs was significantly increased, indicating that AP-3 is involved in MC secretory granule biogenesis. Furthermore, AP-3 knockdown had a selective impact on the secretion of newly formed and newly synthesized mediators. These results show for the first time that AP-3 plays a critical role in secretory granule biogenesis and mediator release in MCs.

## Introduction

Lysosome-Related Organelles (LROs) are functionally diverse cell type-specific subcellular compartments that share many features with lysosomes and are capable of regulated secretion in response to appropriate stimuli [1, 2]. In addition to melanocytes and endothelial cells, LROs are found in many specialized secretory cells of hematopoietic origin and store cell type specific secretory proteins along with lysosomal membrane proteins and hydrolases [3, 4]. LRO biogenesis originates in the biosynthetic pathway where proteins destined for regulated secretion are synthesized in the endoplasmic reticulum and transported sequentially through the Golgi apparatus were they are modified post translationally before reaching the trans-Golgi network (TGN) from which they are sorted either directly to the LROs or indirectly through the plasma membrane and the endosomal-lysosomal system [5, 6].

Protein targeting within the biosynthetic and endosomal-lysosomal system depends on sorting signals that direct their incorporation into transport vesicles for delivery to target organelles [7]. These vesicles display specific coat proteins that aid in the selection of cargo, mechanical bending of the donor membrane and, subsequently, vesicle budding. Clathrin-coated vesicles (CCVs) are the major carriers involved in post-Golgi secretory and endocytic pathways. The formation of CCVs depends on clathrin adaptors that connect clathrin to the donor membrane, select cargo, and recruit accessory proteins, which regulate budding and vesicular trafficking [8, 9]. Among the several classes of clathrin adaptors, the adaptor protein (AP) complexes belong to a family with five members (AP-1, AP-2, AP-3, AP-4 and AP-5), each with distinct membrane localization and functions. They act by recognizing small sequences or motifs which include tyrosine based Yxxϕ (where ϕ is either I, L, M, F, or V) or acidic dileucine motifs [D/E]xxxL[I/L] in the cytosolic tail of cargo proteins [10]. Lysosomal membrane proteins contain one or more of these targeting signals in their cytosolic domains that interact selectively with AP complexes to mediate their incorporation into transport vesicles [8, 11, 12]. The AP-3 complex is composed of two large (δ/β3), a medium (μ3) and a small (σ3) subunit, and is involved in the sorting and trafficking of a subset of transmembrane proteins between tubular endosomes (early endosomes) and/or TGN to late endosomes, lysosomes, and LROs. The importance of AP-3 for LRO biogenesis and regulated secretion is highlighted in Type II Hermansky-Pudlak Syndrome (HPS2), a human autosomal recessive disorder caused by genetic defects in the β3A subunit of AP-3, and in its mouse model Pearl [13, 14]. HPS2 is characterized by oculocutaneous albinism, bleeding disorders and innate immune deficiency. These symptoms are associated with anomalies in LRO biogenesis and secretion in specialized secretory cells including melanocytes, platelets, neutrophils, natural killer cells, and cytotoxic T lymphocytes [15–18].

Mast cells (MCs) are multifunctional immune cells that, in addition to their well-established role in allergic reactions, participate in innate and adaptive immunity, and inflammation among other physiological and pathological processes [19–24]. IgE-dependent MC activation, through aggregation of antigen specific IgE bound to FcεRI on the MC surface, is the most common type of MC activation and triggers the signaling cascade that culminates in the release of three classes of mediators: preformed mediators, which are stored in mast cell secretory granules (LROs); neoformed or lipid mediators, which are newly-formed from membrane lipids; and neosynthesized mediators produced following transcriptional activation [25–27]. In spite of the recognized role of AP-3 in the biogenesis and regulated secretion of LROs in several cell types, the presence of AP-3 and its relevance for these processes in MCs has not been previously investigated. The expression of AP-3 in MCs was confirmed using rat peritoneal mast cells. The involvement of the AP-3 complex in LRO biogenesis and mediator release was then investigated using the RBL-2H3 rat MC line. The AP-3 complex was shown to be associated with the biosynthetic and endocytic pathways in RBL-2H3 MCs and to have a role in regulating secretory granule size. Furthermore, AP-3 knockdown had an impact on RBL-2H3 regulated exocytosis of preformed mediators and also on the secretion of some newly formed and newly synthesized mediators.

## Materials and methods

### Animals

Young (150 g) male and female Wistar rats were used. Animals were housed and experiments were approved and conducted according to the Comissão de Ética em Experimentação Animal, Ribeirão Preto Medical School-USP, Ribeirão Preto, Brazil guidelines (protocol: 032/2007). For all experiments, animals were sacrificed by CO_2_ inhalation.

### Cells

RBL-2H3 rat mast cells [28] were used in this study. Cells were grown as monolayers in Dulbecco’s modified Eagle’s Medium (DMEM) (Invitrogen—Thermo Fisher Scientific, Carlsbad, CA) supplemented with 15% fetal calf serum (Sigma-Aldrich, St. Louis, MO), 0.434 mg/mL glutamine, and an antibiotic-antimycotic mixture containing 100 U/mL penicillin, 100 μg/mL streptomycin, and 0.25 μg/mL amphotericin B (Invitrogen—Thermo Fisher Scientific). The HEK293T cell line [29] was used as a packaging cell line to produce lentiviral particles and was cultured under the same conditions as the RBL-2H3 cells.

### Antibodies

The following primary antibodies were used: mouse mAb anti-δSA4 (10 μg/mL; Developmental Studies Hybridoma Bank, Iowa City, IA; Peden et al, 2004), mouse mAb anti-rat GD1b derived gangliosides—mAb AA4 (5 μg/mL; Clone AR32AA4; BD Pharmingen, San Jose, CA), rabbit polyclonal antibody anti-AP3D1 (1:100; Proteintech Group, Inc., Chicago, IL), mouse mAb anti-p47A (1:500; Clone 26/P47A; BD Transduction Laboratories, San Jose, CA; generously provided by Dr. Gonzalo A. Mardones, Austral University of Chile, Chile), mouse mAb anti-Adaptin γ (1:1000; Clone 88/Adaptin γ; BD Transduction Laboratories), rabbit mAb anti-SNX2 (1:1000; [30]), mouse mAb anti-GM130 (4 μg/mL; Clone 35/GM130; BD Transduction Laboratories), mouse mAb anti-TGN38 (1:800; Clone 2; BD Transduction Laboratories), rabbit polyclonal antibody anti-Cathepsin D (10 μg/mL; Clone IM-16; Calbiochem—Merck KGaA, Darmstadt, Germany), mouse mAb anti-FcεRI alpha subunit conjugated to FITC (15 μg/mL; Clone BC4; generously provided by Dr. Reuben Siraganian, National Institutes of Health—NIDCR, Bethesda, MD), and rabbit polyclonal antibody anti-α/β-tubulin (1:5000; Cell Signaling Technology Inc., Danvers, MA). The following secondary antibodies were used for immunofluorescence and flow cytometry: donkey anti-mouse IgG F(ab)′_2_-Alexa 488 or 594 and donkey anti-rabbit IgG F(ab)′_2_-Alexa 594 or 488 (1:1000; Molecular Probes—Thermo Fisher Scientific). The following secondary antibodies were used for immunoblotting: donkey anti-mouse IgG conjugated to horseradish peroxidase (HRP) and donkey anti-rabbit IgG conjugated to HRP (Jackson ImmunoResearch Laboratories Inc., West Grove, PA). For immunoelectron microscopy goat anti-mouse IgG conjugated to nanogold (Nanoprobes, Yaphank, NY) was used according to manufacturer’s instructions. Mouse IgE anti-TNP ascites fluid was used to sensitize cells before FcεRI stimulation (1:5000; generously provided by Dr. Reuben Siraganian, NIH).

### ShRNA knockdown

MISSION^®^ lentiviral shRNA plasmid vectors encoding AP-3 δ shRNA sequences and a nontargeting shRNA control vector (MISSION TRC2 pLKO.5-puro Non-Mammalian shRNA Control, Catalog No. SHC202), both also containing a puromycin resistance gene, were purchased from Sigma-Aldrich. Two premade lentiviral constructs encoding AP-3 δ shRNAs were used. The shRNAs employed were designed against the following target sequences: Clone 23 (Sh23) 5´-TCCATGTACAGCCGCTCTATCC-3´ and Clone 24 (Sh24) 5´-ACCTGGATGCCTGGATCAATG-3’. Control nontargeting Insert Sequence: 5’- CCGGCAA CAAGATGAAGAGCACCAACTCGAGTTGGTGCTCTTCATCTTGTTGTTTTT– 3’. FuGENE^®^ HD Transfection Reagent (Promega Co., Madison, WI) was used to co-transfect the shRNA vectors with the MISSION^®^ Lentiviral Packing mix (Sigma-Aldrich) into HEK293T packing cells to generate lentiviral particles. RBL-2H3 cells were transduced with control or AP-3 δ lentivirus (Sh23 and Sh24) for 16h at a MOI of 6. The media containing the virus was removed and replaced with fresh DMEM and the cells cultured for 24h before addition of puromycin (1 μg/mL; Sigma-Aldrich) in order to select for cells in which the shRNA was integrated. Real time PCR and immunostaining of permeabilized cells followed by flow cytometry were employed to monitor AP-3 δ mRNA and protein knockdown, respectively; also, immunoblotting of μ3 subunit was employed to confirm AP-3 complex knockdown.

### Real-time PCR

Total RNA was purified from 5.0×10^6^ cells using the Illustra^™^ RNAspin Mini Isolation Kit (GE Healthcare Bio-Sciences, Pittsburgh, PA) according to the manufacturer’s instructions. For cDNA synthesis 5 μg of total RNA was reverse-transcribed using the GoScript^™^ Reverse Transcription System according to the manufacturer’s instructions (Promega). Gene specific primers were used for quantitative PCR analysis. Power SYBR Green PCR Master Mix (Applied Biosystems, Thermo Fisher Scientific, Inc., Foster City, CA) was used with 10 ng of RNA/well of the cDNA product in an ABI 7500 Real Time PCR System (Applied Biosystems, Thermo Fisher Scientific, Inc.). For all RT-PCR analysis, GAPDH mRNA was used to normalize RNA inputs. Primer sequences are as follows:

rat AP3D1 forward (5´-TGTGGAGCTGACAAGACTGG-3´);
rat AP3D1 reverse (5’-ACCAGGTGGGCACTATCAAG-3’);
rat GAPDH forward (5’-GACATGCCGCCTGGAGAAAC-3’);
rat GAPDH reverse (5’-AGCCCAGGATGCCCTTTAGT-3’).

### Flow cytometry

The cells were cultivated (5.0×10^5^ cells) for 16h in Costar T-25 flasks (Corning Life Sciences, Tewksbury, MA), harvested with trypsin-EDTA (Invitrogen—Thermo Fisher Scientific), and rinsed by centrifugation in PBS. To evaluate expression of AP-3 δ subunit, the cells were then fixed for 20 min with 2% paraformaldehyde (Electron Microscopy Sciences, Hatfield, PA) in PBS, washed in PBS and permeabilized with 0.05% saponin in PBS for 15 min, blocked for 30 min at room temperature (RT) in PBS containing 1% BSA (Sigma-Aldrich) and 5 μg/mL normal donkey IgG (Jackson ImmunoResearch). The cells were then incubated with primary antibody for 1h, washed 3 times with PBS and incubated with donkey anti-mouse IgG conjugated to Alexa 488 for 1h at RT and washed 5 times in PBS. After rinsing, the cells were analyzed with a Guava EasyCyte Mini System using Cytosoft Blue software (Guava Technologies, Inc., Hayward, CA). To evaluate FcεRI surface expression, non-permeabilized cells were incubated at 4°C for 1h with mAb BC4-FITC in PBS containing 1% BSA and 5 mg/mL normal donkey IgG, washed in PBS and fixed for 20 min with 2% paraformaldehyde (Electron Microscopy Sciences) before been analyzed with a Guava EasyCyte Mini System using *Cytosoft Blue* software (Guava Technologies, Inc., Hayward, CA).

### SDS-page and immunoblotting

Antibodies to AP-3 μ (mouse mAb anti-p47A) and AP-1 γ (mouse mAb anti-Adaptin γ) subunits where used to evaluate expression of adaptor proteins. Whole cell lysates were mixed with 2X SDS-PAGE sample buffer (4% SDS, 20% Glycerol, 0.12M Tris pH 6.8, and 5% β-Mercaptoethanol), boiled and proteins were separated electrophoretically on 10% polyacrylamide gels and electrotransferred to Hybond nitrocellulose membranes (GE Healthcare Bio-Sciences). After transfer, the membranes were blocked for 1h at RT in TTBS (0.05M Tris—HCl, 0.15M NaCl, pH 7.5, and 0.05% Tween 20) containing 4% BSA and probed for 16h at 4°C with individual primary antibodies, washed in TTBS and incubated with the appropriate anti IgG conjugated to HRP (Jackson ImmunoResearch) for 30 min at RT, washed and developed using chemiluminescence (ECL—GE Healthcare Bio-Sciences). Images were obtained using a Bio-Rad ChemiDoc Imaging System (Bio-Rad Laboratories, Hercules, CA). The mean optical density of the target protein was determined using the Image Lab software (Bio-Rad Laboratories).

### Fluorescence microscopy

Peritoneal cells were obtained by injecting Wistar rats i.p. with 15 mL sterile PBS. The peritoneal wash was collected following laparotomy using a Pasteur pipette. The cells were rinsed twice in PBS and placed on silane-coated Unifrost Microscope Slides (Azer Scientific, Morgantown, PA). The cells were fixed for 20 min with 2% paraformaldehyde (Electron Microscopy Sciences) in PBS, rinsed again, and permeabilized with 0.01% saponin (Sigma-Aldrich) in PBS for 20 min. Next, cells were incubated for 45 min at RT in PBS containing 1% BSA and 5 μg/mL normal donkey IgG (Jackson ImmunoResearch). For double staining with two different mouse monoclonal antibodies, mAb δSA4 and mAb AA4 were fluorescently labeled according to the manufacturer's protocol with the Zenon Alexa Fluor 488 and 594 mouse IgG1 labeling kits (Molecular Probes—Thermo Fisher Scientific), respectively. The cells were then incubated with the directly labeled antibodies for 1h at RT. Cells were then rinsed in PBS and mounted with Fluoromount-G (Electron Microscopy Sciences).

RBL-2H3 cells were plated (5.0×10^4^ cells/coverslip) and cultured for 16h on 13 mm round coverslips. The cells were rinsed in PBS, fixed for 20 min with 2% paraformaldehyde (Electron Microscopy Sciences) in PBS, rinsed again, and permeabilized with 0.01% saponin (Sigma-Aldrich) in PBS for 20 min. Next, cells were rinsed twice in PBS and incubated for 45 min at RT in PBS containing 1% BSA and 5 μg/mL normal donkey IgG (Jackson ImmunoResearch). Cells were then labeled with primary antibodies diluted in PBS containing 1% BSA for 1h at RT. To avoid cross-reactivity, two different antibodies were used to determine the subcellular localization of AP-3. In the double staining of AP-3 with GM130 and TGN38, rabbit polyclonal antibody anti-AP3D1 was used to localize AP-3 since anti-GM130 and anti-TGN38 antibodies were raised in mice. Otherwise, mouse mAb anti-δSA4 was used to localize AP-3 in the double staining of AP-3 with SNX2 and CATD since both anti-SNX2 and anti-CATD antibodies were raised in rabbit. After incubation, cells were rinsed thoroughly in PBS and incubated for 30 min at RT with the appropriate secondary antibodies diluted in PBS. Cells were then rinsed in PBS and mounted with Fluoromount-G (Electron Microscopy Sciences). Cells incubated without primary antibody served as controls and were all negative. All samples were analyzed using a LEICA TCS-NT SP5 laser scanning confocal microscope (Leica Microsystems; Heidelberg, Germany). Colocalization studies were performed on Z-series images by quantitation of Manders’ Colocalization coefficients M1/M2 using Image J software [31] and the colocalization threshold plug-in developed by Tony Collins (Wright Cell Imaging Facility, Toronto, Canada) as previously described [32]. M1 is the percentage of above-background pixels in the green channel that overlap above-background pixels in the red channel. Immunostaining of the δ subunit of AP-3 was considered the green channel and the organelle marker was considered the red channel. The organelle markers were GM130 for *cis*-Golgi, TGN38 for the trans Golgi network, SNX2 for early endosomes, and Cathepsin D for secretory granule protease. A minimum of 8 images was analyzed for each colocalization assay.

### Transmission Electron Microscopy (TEM)

Cells were plated (4.5×10^4^ cells/well) in 6-well tissue culture plates (Corning Life Sciences) and cultured for 2 days before fixation. Media were changed daily before fixation. Cells were rinsed in PBS and fixed by microwave irradiation in 0.05% glutaraldehyde (Electron Microscopy Sciences) plus 4% formaldehyde (Electron Microscopy Sciences) in 0.1 M cacodylate buffer (pH 7.4), containing 0.025% CaCl_2_ for 10s, as previously described [33]. For pre-embedding immunoelectron microscopy cells were rinsed with 50 mM glycine in PBS for 15 min, and blocked for 45 min with 1% BSA in PBS. Cells were then permeabilized with 0.05% saponin in PBS containing 1% BSA for 15 min, incubated with anti-δSA4 for 2h at RT, rinsed, and subsequently incubated with goat anti-mouse IgG conjugated to nanogold (Nanoprobes) for 1h at RT. After rinsing in 3 times with PBS containing 1% BSA with cells were fixed with 2.5% glutaraldehyde in 0.1 M cacodylate buffer for 1h, rinsed twice with 0.1 M cacodylate buffer and 5 times with Milli-Q water. The nanogold was enhanced using GoldEnhance^™^ Electron Microscopy Plus (Nanoprobes) for 6 min according to the manufacturer’s directions. In all TEM experiments cells were post fixed in 1% reduced OsO_4_ (Electron Microscopy Sciences) [34] in 0.1 M cacodylate buffer (pH 7.4) for 2h, rinsed in Milli-Q water, and dehydrated in a graded ethanol series. Cells were removed from the tissue culture plates with propylene oxide and embedded in EMBED 812 (Electron Microscopy Sciences). Thin sections were cut with a diamond knife, mounted on copper grids, and stained for 10 min each in Reynolds’s lead citrate (Reynolds 1963) and 0.5% aqueous uranyl acetate, and examined with a JEOL JEM-100CXII (JEOL Ltd., Tokyo, Japan) transmission electron microscope. For morphometric analysis of mast cell secretory granules images of a minimum of 30 cells from each condition were analyzed using Image J software [31]. For area measurements, the secretory granules were manually selected with the freehand selection tool and the area measurements were calculated using the ROI manager tool. The selection criteria for the RBL-2H3 secretory granules were based on following morphological features: membrane limited organelles displaying internal membrane vesicles interspace with dense membranous material and electrontranslucent areas. Double membrane compartments were not selected to avoid confusion with mitochondria.

### Scanning Electron Microscopy (SEM)

Cells were plated on 13 mm round coverslips (5.0×10^4^ cells/coverslip) and sensitized or not with IgE anti-TNP ascites fluid in the culture medium and incubated for 16h followed or not by stimulation with 50 ng/mL of DNP_48_-HSA (Sigma-Aldrich) for 15 min. Cells were rinsed in warm PBS (37°C) and fixed with 2% glutaraldehyde (Electron Microscopy Sciences) in warm PBS for 2h at RT. Cells were post fixed in 1% OsO_4_ (Electron Microscopy Sciences) for 2h, rinsed in Milli-Q water, incubated with a saturated solution of thiocarbohydrazide (Electron Microscopy Sciences), followed by 1% OsO_4_. This step was repeated once. The cells were dehydrated in a graded series of ethanol and critically point-dried with liquid CO_2_ in a Tousimis Autosandri-810 (Tousimis Research Co., Rockville, MD), mounted on aluminum stubs with silver paint (Electron Microscopy Sciences), and coated with gold in a BAL-TEC SCD 050 Sputter Coater (BAL-TEC). Samples were examined with a JEOL JSM-6610 LV scanning electron microscope (JEOL, Ltd.; Tokyo, Japan).

### β-hexosamidase assay

Mast cell degranulation was assessed by measuring the activity of released β-hexosaminidase after FcεRI stimulation. Cells were plated (3.0×10^4^ cells/well) in a 96 well tissue culture plate (Costar-Corning Inc.), sensitized or not with IgE anti-TNP ascites fluid in the culture medium and incubated for 16h followed or not by stimulation with 50 ng/mL of DNP_48_-HSA (Sigma-Aldrich) for 45 min. β-hexosaminidase release was determined as previously described [35]. β-hexosaminidase activity was quantified in the supernatants and cell lysates by spectrophotometric analysis of hydrolysis of 4-Nitrophenyl N-acetyl-β-D-glucosaminide (Sigma-Aldrich). β-hexosaminidase release was calculated as the percentage of β-hexosaminidase activity measured in the supernatants relative to the total amount of β-hexosaminidase activity measured in the supernatant and cells.

### Lipid mediator release assay

Cells were plated (1.0×10^5^ cells/well) in a 24 well tissue culture plate (Corning Life Sciences) sensitized or not with IgE anti-TNP ascites fluid (1:5000 dilution) in the culture medium and incubated for 16h followed or not by stimulation with 50 ng/mL of DNP_48_-HSA (Sigma-Aldrich) for 30 min. PGD2 and LTC4 in culture supernatants were analyzed using EIA kits (Cayman Chemical, Ann Arbor, MI).

### Cytokine release assay

Cells were plated (1.0×10^5^ cells/well) in a 24 well tissue culture plate (Corning Life Sciences) sensitized with IgE anti-TNP ascites fluid (1:5000 dilution) in the culture medium and incubated for 16h followed by stimulation with 50 ng/mL of DNP_48_-HSA (Sigma-Aldrich) for 1h. Culture supernatants were discarded and the cells were washed and incubated with fresh media for an additional 23h. The supernatants were collected and released cytokines were analyzed using the *Proteome Profiler Rat Cytokine Array Kit*, *Panel A* (R&D Systems, Inc. Minneapolis, MN) according to the manufacturer’s instructions. Briefly, supernatants were mixed with a cocktail of biotinylated detection antibodies and then incubated with the membrane containing immobilized antibodies for 29 rat cytokines. Bound protein was detected with streptavidin conjugated to HRP. Membranes were washed and developed using ECL^™^ Western Blotting Detection Reagent RPN2106 (GE Healthcare).

### Statistics

Results were analyzed using GraphPad Prism (GraphPad Software, Inc., La Jolla, CA) and expressed as mean ± SD. In the colocalization experiments, statistical differences were assessed by a two-tailed unpaired t test with Welch's correction. In all shRNA experiments differences between groups were assessed by one-way ANOVA with Dunnett´s post-test and all groups were compared with the shRNA control group; A *p*<0.05 (*) was considered significant.

## Results

### The AP-3 complex is expressed in rat peritoneal mast cells

**Fig 1:**
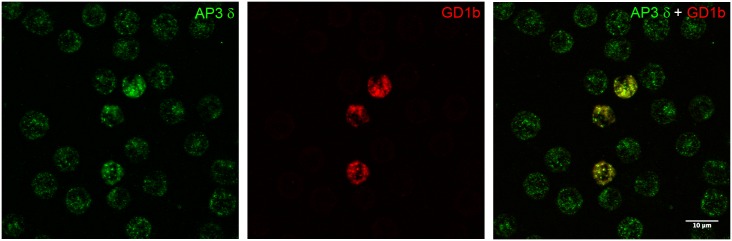
AP-3 is expressed in rat peritoneal mast cells. AP-3 was distributed in a punctate fashion in the cytoplasm of cells from the rat peritoneal lavage. Double labeling of the peritoneal lavage for AP-3 and the MC specific GD1b derived gangliosides revealed that the MCs were prominently stained for AP-3. Anti-AP-3 δ antibody (mAb anti-δSA4) was fluorescently labeled with the Zenon Alexa Fluor 488 (Green) and anti-MC specific GD1b derived gangliosides (mAb AA4) was fluorescently labeled with the Zenon Alexa Fluor 594 (Red). Bar = 10 μm.

Since the presence of AP-3 has not been previously reported in MCs, the expression of AP-3 in rat peritoneal mast cells was investigated by direct immunofluorescence. The peritoneal lavage from Wistar rats was immunostained with antibodies to the δ subunit of AP-3. In order to conclusively identify MCs, the peritoneal lavage was also immunostained for the MC specific gangliosides derived from GD1b. These rodent mast cell specific gangliosides are expressed in both immature and mature rat mast cells [36, 37]. AP-3 displayed a punctate staining pattern throughout the cytoplasm of all the cells in the peritoneal lavage. Furthermore, the MCs appeared to be more intensely stained (Fig 1), thus demonstrating that AP-3 is present in MCs *ex vivo*.

### The AP-3 complex colocalizes with the biosynthetic and endocytic pathways in RBL-2H3 cells

**Fig 2:**
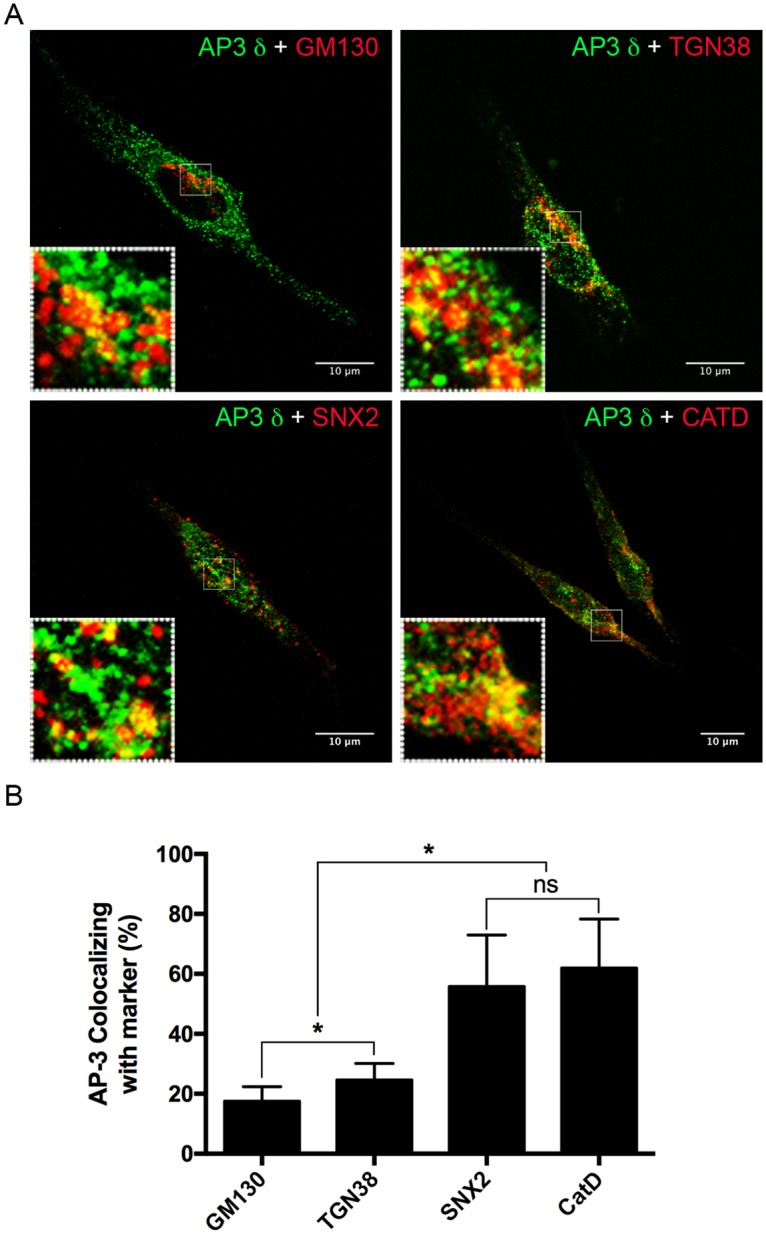
AP-3 colocalizes with markers of the biosynthetic and endocytic pathways. (A) AP-3 was distributed in a punctate fashion in the cytoplasm of the RBL-2H3 cells. Double labeling of AP-3 with anti-GM130 (*cis*-Golgi), anti-TGN38 (TGN), anti-SNX2 (early endosomes) or anti-Cathepsin D (secretory granules) showed a partial colocalization. The rabbit polyclonal antibody anti-AP3D1 was used in the double staining of AP-3 with GM130 and TGN38 (upper panels) and the mouse mAb anti-δSA4 was used in the double staining of AP-3 with SNX2 and CATD (lower panels). Anti-AP-3 δ antibodies were detected with secondary antibodies conjugated with Alexa-488 (green); anti-GM130, anti-TGN38, anti-SNX2, and anti-CATD were detected with secondary antibodies conjugated to Alexa 594 (red). Bar = 10 μm. (B) Manders’ colocalization coefficient values are expressed as the percentage of AP-3 that colocalized with the organelle markers. Data is expressed as the mean ± SD of colocalization analysis of at least eight individual images from a total of three independent experiments. ns: not significant; *: p ≤ 0.05.

In order to facilitate the investigation of the role of AP-3 in MC regulated secretion, the rat mast cell line RBL-2H3 was used in all further experiments. The subcellular localization of AP-3 in these cells was analyzed by immunostaining with antibodies to the δ subunit of AP-3 as well as markers for components of the secretory pathway (Fig 2A). AP-3 displayed a punctate staining pattern throughout the cytoplasm similar to that seen for peritoneal mast cells, and partially colocalized with markers for the *cis*-Golgi saccules, TGN, tubule-vesicular early endosomes and secretory granules. An analysis of the percentage of colocalization of AP-3 with the various markers showed that the colocalization was lowest in the *cis*-Golgi saccules but increased significantly in the TGN, early endosomes and secretory granules (Fig 2B).

**Fig 3:**
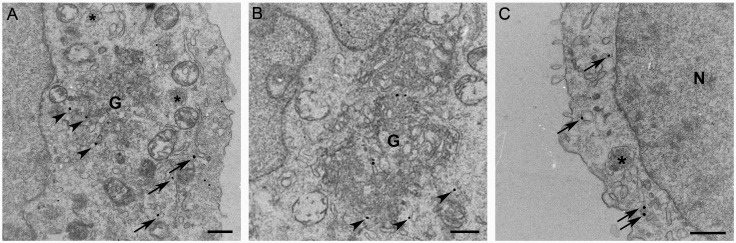
AP-3 is associated with membrane bound organelles in the biosynthetic and endocytic pathway. By immunoelectron microscopy, AP-3 was localized on cytoplasmic side of vesicles and tubular structures in close proximity to the Golgi complex (A and B) (arrowheads) and on tubule-vesicular endosomal membranes (A and C) adjacent to the plasma membrane (arrows). Bar = 0.5 μm. N: Nucleus; G: Golgi; *: Secretory Granule.

By immunoelectron microscopy, AP-3 was found associated with the cytoplasmic face of vesicles and tubular structures in close proximity to the Golgi complex (Fig 3A and 3B). AP-3 was also associated with vesicles and tubular endosomal structures near the plasma membrane (Fig 3A and 3C). The association of AP-3 with the TGN and tubule-vesicular endosomes indicates that in MCs, AP-3 dependent sorting of proteins to secretory granules originates in these organelles.

### AP-3 δ knockdown destabilizes the AP-3 complex

**Fig 4:**
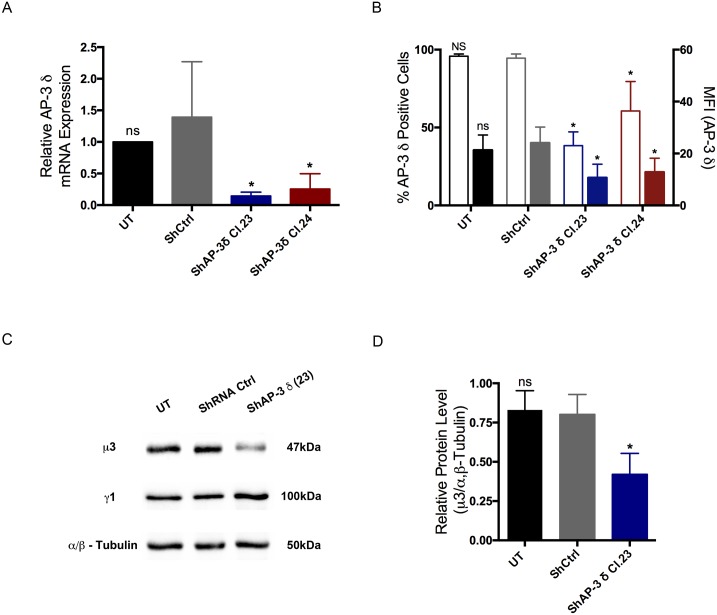
ShRNA mediated depletion of AP-3 δ subunit destabilizes the AP-3 adaptor complex in RBL-2H3 mast cells. RBL-2H3 cells were transduced with lentiviral particles expressing shRNAs against the δ subunit of the AP-3 complex (ShAP-3 δ Cl.23 and ShAP-3 δ Cl.24) or with non-targeting shRNA control (ShCtrl). (A) By quantitative RT-PCR, RBL-2H3 cells transduced with AP-3 δ shRNAs had an average reduction of 80% in AP-3 δ mRNA expression when compared to shRNA control cells (ShCtrl). (B) By FACS analysis of permeabilized cells immunostained with anti-δSA4 antibody, AP-3 δ protein levels were reduced by approximately 50% when compared to ShCtrl cells. Open Bars: % of AP-3 δ positive cells; Colored Bars: Mean Fluorescence Intensity (MFI). (C) Western blot analysis showed that in RBL-2H3 cells knocked down for AP-3 δ there was a reduction in AP-3 μ3 expression while AP-1 γ protein levels were unaltered. Representative Western blot images. (D) Ratio of AP-3 μ3 to α/β tubulin. The 50% reduction in AP-3 μ3 expression was equivalent to that seen for AP-3 δ. The AP-3 μ3 subunit was immunolabeled with the mAb anti-p47A and the AP-1 γ was labeled with the mAb anti-Adaptin γ. Data is expressed as the mean ± SD of at least three independent experiments. Statistical difference in comparison to shRNA control transduced cells (ShCtrl). ns: not significant; *: p ≤ 0.05; UT: untransduced cells.

To assess a possible role for AP-3 in MC regulated exocytosis, the expression of AP-3 was knocked down in RBL-2H3 mast cells. RBL-2H3 cells were transduced with lentiviral particles encoding for two different shRNAs against the δ subunit of AP-3. Quantitative RT-PCR showed an approximately 80% decrease in AP-3 δ mRNA expression compared to cells expressing a nontargeting shRNA (Fig 4A). Flow cytometry analysis showed a 50% reduction in AP-3 δ protein levels compared to control cells (Fig 4B). Western blot analysis using an antibody specific to the μ3 subunit of AP-3 showed that AP-3 δ knockdown leads to an equivalent reduction (50%) in μ3 levels confirming that the whole complex was destabilized (Fig 4C and 4D). Moreover, AP-3 δ knockdown did not affect the levels of AP-1 γ expressed in MCs (Fig 4C).

### ShRNA mediated depletion of AP-3 adaptor complex did not interfere with FcεRI expression or the morphological changes associated with MC activation

**Fig 5:**
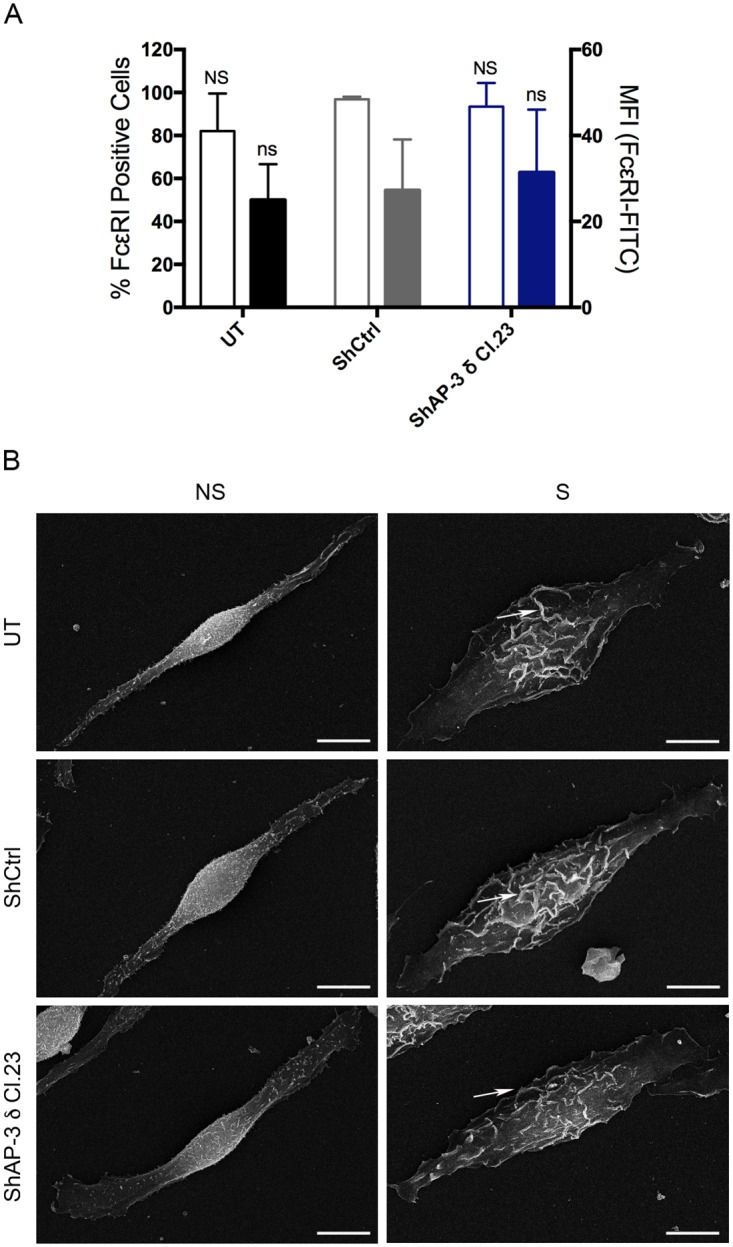
ShRNA mediated depletion of AP-3 does not interfere with FcεRI surface expression or the morphological changes characteristic of MC activation. (A) By FACS analysis, there were no significant differences in expression of FcεRI on the surface of RBL-2H3 cells transduced with AP-3 δ shRNA (ShAP-3 δ Cl.23) and shRNA control (ShCtrl) or untransduced cells (UT). Open Bars: % of FcεRI positive cells; Colored Bars: Mean Fluorescence Intensity (MFI). Data is expressed as the mean ± SD of three independent experiments. Statistical differences are in comparison to shRNA control transduced cells (ShCtrl). ns: not significant. (B) By scanning electron microscopy AP-3 knockdown did not interfere with cell spreading or ruffling (arrows) of RBL-2H3 cells activated via FcεRI. NS: non-stimulated; S: stimulated via FcεRI.

FcεRI mediated MC activation is the best characterized pathway of mast cell activation, which is crucial for the regulated secretion of MC mediators. Therefore, it was of interest to verify that shRNA mediated AP-3 knockdown did not interfere with key features of FcεRI activation such as FcεRI surface expression and activation induced membrane ruffling. Knockdown of AP-3 δ did not alter the expression of FcεRI on the surface of RBL-2H3 cells, as detected by flow cytometry analysis (Fig 5A). Furthermore, shRNA mediated AP-3 knockdown did not affect the cell spreading and surface ruffling that are characteristic of mast cell activation (Fig 5B).

### The AP-3 complex is critical for regulated secretion of preformed mediators

**Fig 6:**
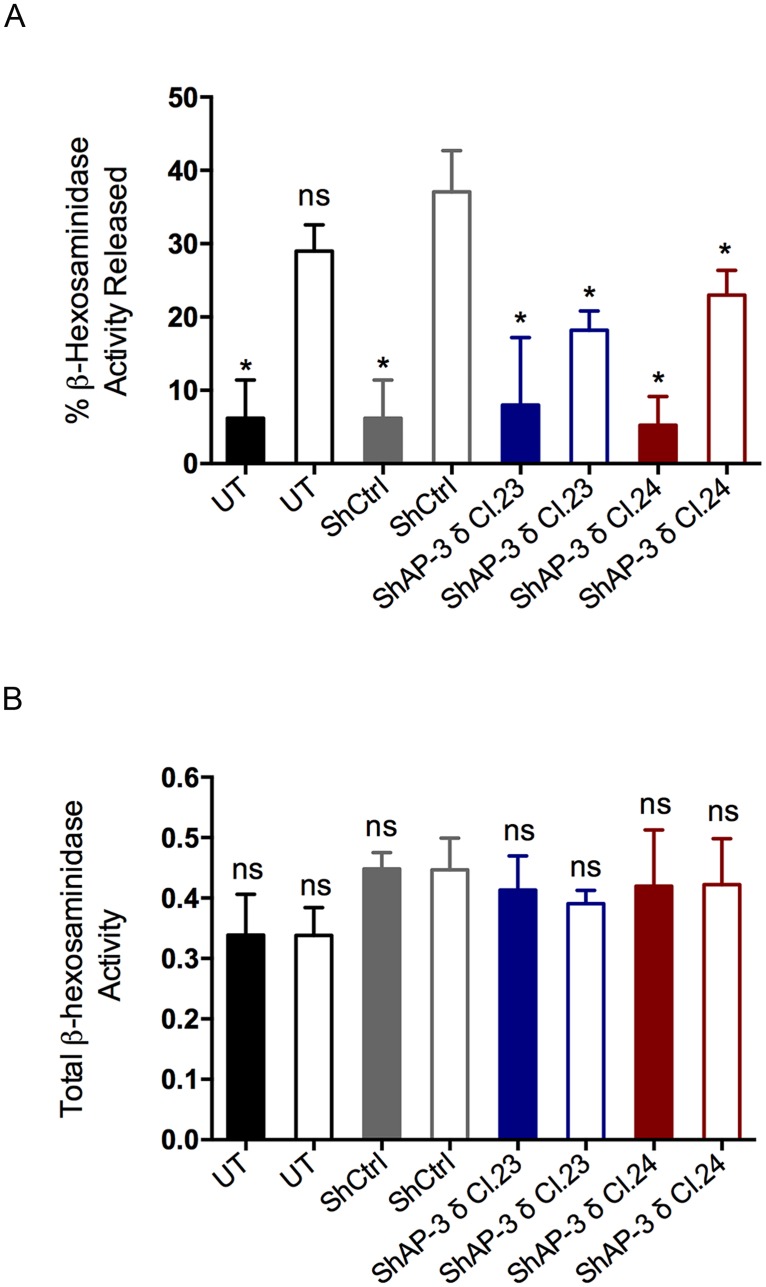
AP-3 knockdown resulted in decreased β-hexosaminidase release in FcεRI stimulated mast cells. For stimulation via FcεRI, RBL-2H3 cells were sensitized with IgE anti-DNP and stimulated with DNP_48_-HSA (50 ng/mL) for 45 min. The activity of released and total β-hexosaminidase was determined for stimulated (Open Bars) and non-stimulated cells (Colored Bars). (A) FcεRI stimulated RBL-2H3 cells transduced with AP-3 δ shRNAs (ShAP-3 δ Cl.23 and Cl.24) had an average 45% reduction in the release of β-hexosaminidase activity when compared to shRNA control cells (ShCtrl). (B) Total β-hexosaminidase activity levels were unaltered in AP-3 knockdown cells when compared to ShCtrl cells. Data is expressed as the mean ± SD of three independent experiments. Statistical differences are in comparison to stimulated shRNA control transduced cells (ShCtrl). ns: not significant; *: p ≤ 0.05; UT: untransduced cells. Black Line Bars: UT; Gray Line Bars: ShCtrl; Blue Line Bars: ShAP-3δ Cl.23; and Red Line Bars: ShAP-3δ Cl.24.

Although AP-3 knockdown did not interfere with FcεRI expression or the morphological changes induced by activation via FcεRI, it was of interest to determine if the secretion of preformed MC mediators was affected following FcεRI stimulation. The 50% reduction in AP-3 protein levels was sufficient to significantly affect MC regulated secretion of β-hexosaminidase (Fig 6A). Knockdown of the δ subunit of AP-3 caused an approximately 45% reduction in release of β-hexosaminidase activity, for both δ shRNAs tested, in comparison to RBL-2H3 cells transduced with control shRNA. Furthermore, this observed decrease was not a consequence of reduced cellular levels of β-hexosaminidase since the total enzyme activity in AP-3 depleted cells was not significantly different from the total enzyme activity present in control cells (Fig 6B). This result indicates that AP-3 plays a critical role in regulated exocytosis in MC.

### AP-3 knockdown leads to an enlargement of MC secretory granules

**Fig 7:**
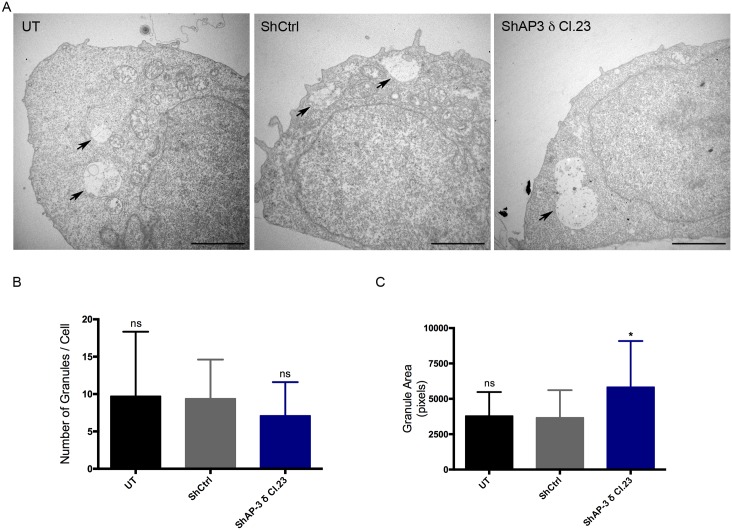
AP-3 knockdown leads to enlarged MC secretory granules. RBL-2H3 MCs were transduced or not with shRNAs and analyzed by transmission electron microscopy. (A) The area of the secretory granules was increased in the AP-3 knockdown cells (ShAP-3 δ Cl.23) when compared to shRNA control cells (ShCtrl). UT: untransduced cells; ShCtrl: shRNA control transduced cells; and ShAP-3 δ Cl.23: shRNA AP-3 δ Cl.23 transduced cells. The arrows indicate secretory granules. Bar = 5 μm. The graphs show the quantification of secretory granules numbers per cell (B) and granule area expressed in pixels (C). Data is expressed as the mean ± SD from the image analysis of at least 30 individual cells from a total of three independent experiments. shRNA AP-3 δ Cl.23 transduced cells were compared to shRNA control transduced cells (ShCtrl). ns: not significant; *: p ≤ 0.05.

Since AP-3 knockdown affected release of preformed mediators that are stored in secretory granules, it was of interest to evaluate secretory granule morphology. MC granules were evaluated by electron microscopy in order to investigate the involvement of AP-3 in the biogenesis of secretory granules. In RBL-2H3 MCs secretory granules are membrane-limited organelles displaying internal membrane vesicles interspersed with electron dense and electron lucent areas. AP-3 knockdown did not interfere with the overall ultrastructure of the secretory granules in these cells or affect the average number of granules per cell. However, a significant increase in the area of the MC granules was observed in AP-3 knockdown MCs (Fig 7A–7C). This increase in granule area further indicates that AP-3 is involved in MC secretory granule biogenesis.

### AP-3 knockdown influences newly formed mediator release

**Fig 8:**
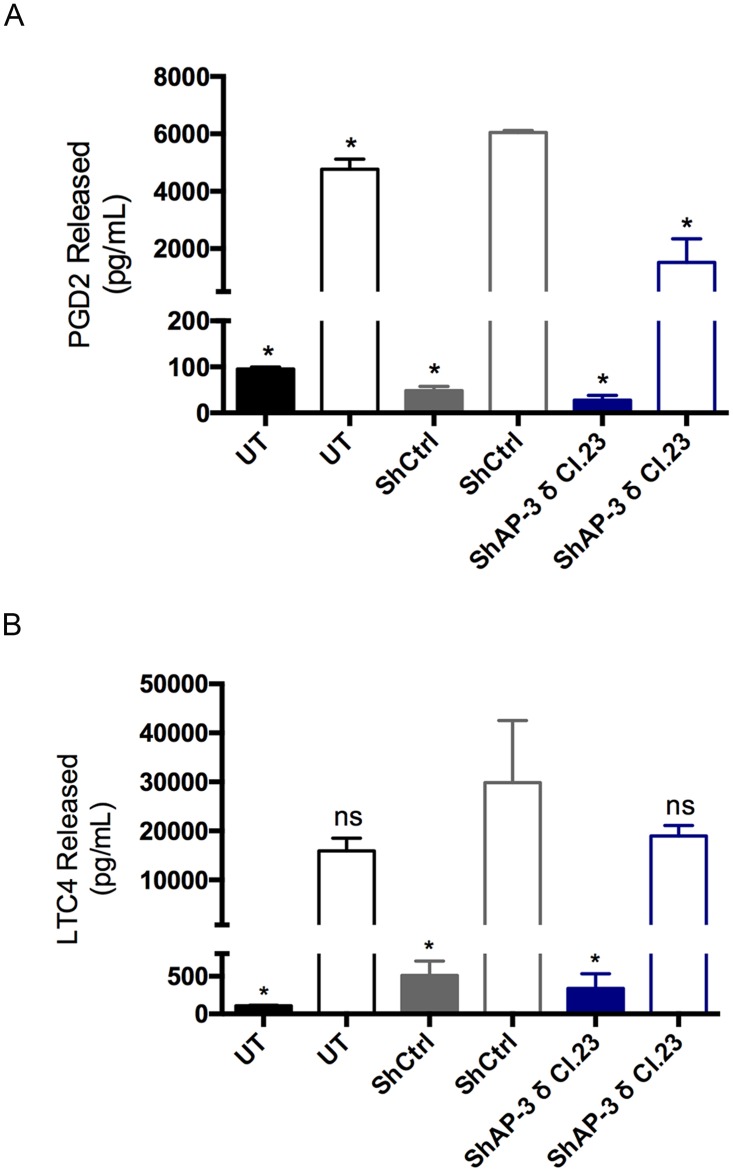
AP-3 knockdown affects newly formed mediator release. For stimulation via FcεRI, RBL-2H3 cells were sensitized with IgE anti-TNP and stimulated with DNP_48_-HSA (50 ng/mL). Lipid mediators were measured, 30 min after stimulation, in the culture supernatant by EIA. Prostaglandin D2 release (A) was decreased in the AP-3 knockdown cells (ShAP-3 δ Cl.23) while release of leukotriene C4 (B) was unaltered when compared to shRNA control cells (ShCtrl). Data is expressed as the mean ± SD of two independent experiments. Statistical differences are in comparison to shRNA control transduced cells (ShCtrl). ns: not significant; *: p ≤ 0.05; UT: untransduced cells.

In addition to the regulated release of preformed mediators, mast cell activation leads to the *de novo* synthesis of lipid mediators, such as prostaglandins and leukotrienes, which are immediately formed after MC activation. The functional impact of AP-3 knockdown on the secretion of newly formed mediators following FcεRI stimulation was investigated. Release of the lipid mediator prostaglandin D2 (PGD2) was decreased after FcεRI activation in AP-3 knockdown MCs (Fig 8A) while leukotriene C4 (LTC4) release was not altered (Fig 8B). Therefore, AP-3 knockdown selectively affects lipid mediator release.

### AP-3 knockdown affects release of newly synthesized mediators

Mast cell activation stimulates the synthesis and release of newly synthesized mediators such as cytokines and growth factors. Cytokines and growth factors are transcriptionally upregulated after MC activation and released by constitutive secretion. To investigate release of newly synthesized mediators IgE sensitized MCs were stimulated and after 1h the culture supernatants were discarded to exclude released preformed cytokines. After 23h of incubation with fresh media the supernatants were analyzed using the *Proteome Profiler Rat Cytokine Array Kit (Panel A)* as described in Materials and Methods. Of the 29 cytokines analyzed 6 had their secretion significantly increased in AP-3 knockdown MCs when compared to ShCtrl cells (Table 1). The secretion of another 21 cytokines was also increased in AP-3 knockdown cells, although this increase was not significant. These results show the knockdown of AP-3 also influences the release of newly synthesized mediators.

**Table 1:** AP-3 knockdown affects the release of newly synthesized mediators.

	MEAN PIXEL DENSITY ± SD		
Cytokines	UT	ShCtrl	ShAP-3 δ Cl.23
CINC-1*	1.44 ±0.17	1.36 ±0.11	3.10 ±0.45
CINC-2α/β*	0.60 ±0.27	0.41 ±0.13	1.88 ±0.36
CINC-3*	1.01 ±0.20	1.00 ±0.14	1.67 ±0.15
CNTF	1.03 ±0.34	0.68 ±0.34	1.37 ±0.35
Fractalkine/CX3CL1	0.81 ±0.34	0.68 ±0.25	1.50 ±0.35
GM-CSF*	0.97 ±0.28	0.73 ±0.09	1.78 ±0.23
sICAM-1/CD54	9.20 ±1.08	9.48 ±2.84	13.11 ±2.48
IFN-γ*	1.96 ±0.41	0.85 ±0.12	2.64 ±0.36
IL-1α/IL-1F1	2.08 ±0.43	1.87 ±0.26	2.74 ±0.40
IL-1β/IL-1F2*	1.20 ±0.23	1.21 ±0.23	2.28 ±0.15
IL-1ra/IL-1F3	1.18 ±0.19	1.05 ±0.17	1.80 ±0.29
IL-2	1.61 ±0.36	1.39 ±0.45	1.53 ±0.40
IL-3	5.04 ±1.67	3.97 ±0.65	5.77 ±1.23
IL-4	4.04 ±0.68	4.27 ±0.83	5.78 ±1.46
IL-6	1.23 ±0.28	0.52 ±0.15	1.13 ±0.10
IL10	1.48 ±0.29	1.29 ±0.24	1.71 ±0.13
IL-13	30.73 ±14.07	27.49 ±11.10	35.35 ±14.38
IL-17	2.44 ±0.67	2.31 ±0.82	3.25 ±0.53
IP-10/CXCL10	1.23 ±0.21	1.20 ±0.30	1.71 ±0.34
LIX	1.92 ±0.34	1.71 ±0.64	1.51 ±0.53
L-Selectin/CD62L	2.21 ±0.27	1.90 ±0.40	2.20 ±0.33
MIG/CXCL9	1.71 ±0.44	1.38 ±0.41	1.98 ±0.24
MIP-1α/CCL3	1.29 ±0.40	0.98 ±0.29	1.29 ±0.19
MIP-3α/CCL20	1.37 ±0.32	1.39 ±0.10	1.28 ±0.08
RANTES/CCL5	2.55 ±0.21	2.21 ±0.65	2.87 ±0.24
Thymus Chemokine/CXCL-7	44.44 ±5.55	52.04 ±2.25	61.18 ±8.25
TIMP-1	1.31 ±0.18	0.88 ±0.22	1.41 ±0.26
TNF-α	1.20 ±0.07	0.52 ±0.15	0.74 ±0.20
VEGF	26.95 ±1.17	27.69 ±4.16	28.08 ±5.55

The Proteome Profiler^™^ - Rat Cytokine Array Panel A—was used to simultaneously assess the relative levels of 29 cytokines released in the supernatant of FcεRI stimulated MCs. Data is expressed as the mean pixel density ± SD of three independent experiments. The mean pixel densities of spots for each cytokine in the membrane array were quantified using Image J. Cytokines whose secretion was significantly increased in AP-3 knockdown MCs are shaded in gray.

*: p ≤ 0.05 in comparison to ShCtrl; UT: untransduced cells; ShCtrl: shRNA control transduced cells; and ShAP-3 δ Cl.23: shRNA AP-3 δ Cl.23 transduced cells.

## Discussion

The present investigation demonstrates, for the first time, that the AP-3 adaptor complex is present in rat MCs and that it is associated with both the biosynthetic and endocytic pathways of RBL-2H3 MCs. Additionally, AP-3 was shown to be important for MC regulated exocytosis of preformed mediators and to have a role in regulating MC secretory granule size. Furthermore, the reduced expression of AP-3 had an impact on the secretion of some newly formed and newly synthesized mediators.

The present study shows an association of AP-3 with early endosomes as well as with the TGN. In spite of its recognized involvement in LRO biogenesis, the subcellular localization of AP-3 has long been a matter of debate. Several studies using immunofluorescence and immunogold labeling of AP-3 in different cell types have yielded conflicting results with respect to the localization of AP-3, suggesting a role for AP-3 in protein sorting either in the TGN or in endosomal compartments [38–43]. However, the association of AP-3 with one set of organelles does not exclude its involvement with another set. In the regulated secretory pathway, proteins destined for secretion are targeted to secretory granules either directly from the TGN or indirectly, via the endosomal system [6]. Both the biosynthetic and endocytic pathways were previously shown to be involved in LRO biogenesis in MCs. Some studies in MCs have demonstrated that the sorting of lysosomal proteases relies mainly on their glycosylation in the Golgi complex and subsequent targeting of these proteins by the mannose-6-phosphate receptor to MC secretory granules [44, 45]. In contrast, other studies have demonstrated that internalized cargo can also be delivered to MC secretory granules [46, 47]. The results of the present study provide evidence for a role of AP-3 in sorting and trafficking from both the TGN and endosomes in RBL-2H3 MCs.

Knockdown of AP-3 was used to functionally study AP-3 in RBL-2H3 MCs. ShRNA mediated knockdown of AP-3 δ subunit in MCs compromised the stability of the μ3 subunit indicating the AP-3 complex was destabilized. This observation is in agreement with previous studies showing that in the absence of one subunit the whole adaptor complex becomes unstable and the levels of the other subunits are diminished [48–51].

Morphometric analysis of RBL-2H3 secretory granules by electron microscopy showed that AP-3 knockdown caused a significant increase in the size of secretory granules, suggesting a role for AP-3 in RBL-2H3 MC secretory granule biogenesis. This finding agrees with previous studies that found a similar phenotype in AP-3 deficient neurons, neuroendocrine cells, cytotoxic T lymphocytes, and platelets [15, 18, 51–53]. One possible explanation for the increased granule size is that AP-3 is involved in secretory granule maturation. In RBL-2H3 MCs, knockdown of Synaptotagmin III (Syt III) leads to an increase in secretory granule size. The authors suggest that this is the result of deficiencies in the retrieval and recycling of granule proteins during granule maturation [54]. We analyzed the protein sequence of Syt III using the Eukaryotic Linear Motif database. This analysis indicated the presence of three tyrosine based motifs and one dileucine based motif in its cytosolic portion (data not shown), which could implicate AP-3 in Syt III sorting to the regulated secretory pathway. Further studies are necessary to confirm this hypothesis and to elucidate the mechanisms by which AP-3 acts in secretory granule biogenesis.

The present investigation indicates that AP-3 plays a role in regulated secretion in RBL-2H3 MCs. After stimulation via FcεRI, MCs deficient in AP-3 released significantly less of the preformed mediator β-hexosaminidase, although the total amount of β-hexosaminidase was the same as that present in the control cells. Additionally, the reduced release of β-hexosaminidase was not a consequence of changes in FcεRI expression on the cell surface. The AP-3 knockdown RBL-2H3 cells also underwent morphological changes characteristic of FcεRI activation. Our results are consistent with previous reports showing reduced secretion of lysosomal hydrolases by platelets from Pearl mice, which are deficient in the AP-3 β3A subunit [17, 55, 56]. Some proteins, which are important for MC regulated secretion, were shown, in other cell types, to contain cytoplasmic sequences required for their correct sorting to secretory granules through an AP-3 dependent pathway [57–59]. SNARE proteins mediate membrane fusion and are crucial for exocytosis. In the plasma membrane, the SNAREs involved in MC degranulation are Syntaxin 4 and SNAP23, whereas in the granule membrane the SNAREs VAMP7 and VAMP8 facilitate membrane fusion [60, 61]. In other cell types, AP-3 was shown to interact with VAMP7 and target it to lysosomes and late endosomes [59]. Therefore, incorrect targeting of VAMP7 in AP-3 deficient MC could interfere with the granule’s ability to fuse with the plasma membrane and release its contents.

AP-3 can also influence the secretion of newly formed mediators. AP-3 deficient RBL-2H3 MCs released less PGD2, but release of LTC4 was unaltered. PGD2 and LTC4 are the main MC eicosanoids produced upon stimulation. Following FcεRI activation, cPLA_2_ is translocated to membranes of intracellular compartments such as the endoplasmic reticulum, nuclear envelope, phagosomal membranes, and to lipid bodies where it liberates arachidonic acid (AA). Distinct metabolic pathways, involving different sets enzymes, are activated to sequentially convert AA to prostaglandins or leukotrienes [62, 63]. Activation of different signaling pathways in MCs can lead to differential release of specific mediators. When MCs are activated by crosslinking GD1b derived gangliosides on their surface, the release of prostaglandins is stimulated, but not leukotrienes [64]. In spite of the fact that lipid mediators do not rely on vesicular traffic to be secreted, compartmentalized synthesis depends on the translocation and assembly of specific enzymatic complexes necessary for the synthesis of a particular mediator [65]. Moreover, the release of the negatively charged lipid mediators to the extracellular environment relies on organic anion transporters of the ATP-binding cassette type III family, known as MRPs (multidrug-resistant related proteins) and SLCs (soluble carrier super family) [66–69]. This dependence on a membrane localized anionic transporter may account for differences in the release of specific mediators. Our data suggest that AP-3 indirectly influences PGD2 secretion by interfering with either synthesis or transporter mediated secretion.

AP-3 deficiency also resulted in increased secretion of several newly synthesized cytokines, which are transcriptionally upregulated after MC activation. In contrast to lipid mediators, cytokines are synthesized in the endoplasmic reticulum and travel through the secretory pathway by means of vesicular transport and are presumably released by constitutive secretion [27, 70]. The molecular mechanisms involved in cytokine secretion are still greatly unexplored and further investigation is necessary to clarify how AP-3 influences neosynthesized mediator release.

Taken together the results of the present study show that AP-3 activity is key to secretion of RBL-2H3 MC mediators, particularly for the biogenesis and regulated release of preformed mediators. In spite of significant advances in the knowledge of MC biology, the understanding of how MC mediators are selectively sorted and released from distinct secretory pathways and the proteins involved are largely unknown. The current investigation provides evidence of the participation and importance of AP-3 for these processes in rat mast cells.
